# Supplementary material for: Predicting visual function by interpreting a neuronal wiring diagram
Source: Nature. 2024 Oct 2;634(8032):113–23. doi: 10.1038/s41586-024-07953-5 (PMC11446822; doi:10.1038/s41586-024-07953-5)
Supplement: Supplementary file 6 — CRF and ERF predictions for individual TmY4 and TmY9 cells. Analogous to Supplementary Data 3, but for TmY target types. Shown are the top four monosynaptic pathways, the strongest pathway passing through each of the top ten intermediary types (ranking from Extended Data Fig. 7), and the trisynaptic pathway Tm1–TmY–Dm3–TmY (see the section entitled Prediction of spatial normalization). [file 41586_2024_7953_MOESM6_ESM.zip › DataS4/TmY9qΓèÑ/720575940617059670.pdf]

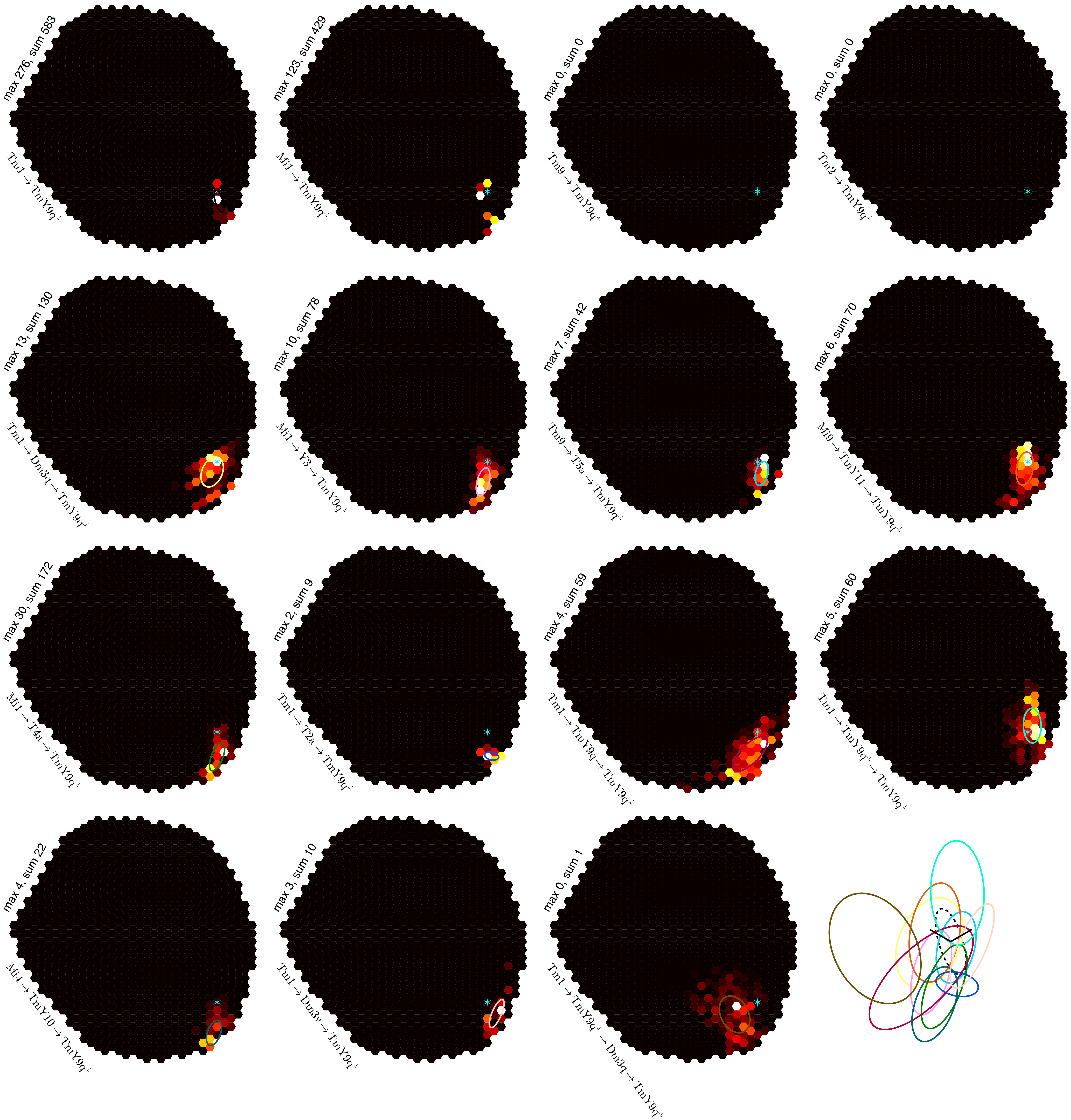
max 276, sum 583
Tm1 → TmY9q⊥
max 123, sum 429
Mi1 → TmY9q⊥
max 0, sum 0
Tm9 → TmY9q⊥
max 0, sum 0
Tm2 → TmY9q⊥
max 13, sum 130
Tm1 → Dm3q → TmY9q⊥
max 10, sum 78
Mi1 → Y3 → TmY9q⊥
max 7, sum 42
Tm9 → T5a → TmY9q⊥
max 6, sum 70
Mi9 → TmY11 → TmY9q⊥
max 30, sum 172
Mi1 → T4a → TmY9q⊥
max 2, sum 9
Tm1 → T2a → TmY9q⊥
max 4, sum 59
Tm1 → TmY9q → TmY9q⊥
max 5, sum 60
Tm1 → TmY9q⊥ → TmY9q⊥
max 4, sum 22
Mi4 → TmY10 → TmY9q⊥
max 3, sum 10
Tm1 → Dm3v → TmY9q⊥
max 0, sum 1
Tm1 → TmY9q⊥ → Dm3q → TmY9q⊥
